# Bone morphogenetic protein 4 in perivascular adipose tissue ameliorates hypertension through regulation of angiotensinogen

**DOI:** 10.3389/fcvm.2022.1038176

**Published:** 2022-11-14

**Authors:** Wen-Juan Mu, Yan-Jue Song, Li-Jie Yang, Shu-Wen Qian, Qi-Qi Yang, Yang Liu, Qi-Qun Tang, Yan Tang

**Affiliations:** Key Laboratory of Metabolism and Molecular Medicine of the Ministry of Education, Department of Biochemistry and Molecular Biology of School of Basic Medical Sciences and Department of Endocrinology and Metabolism of Zhongshan Hospital, Fudan University, Shanghai, China

**Keywords:** perivascular adipose tissue, metabolism, hypertension, renin-angiotensin-aldosterone system (RAAS), BMP4

## Abstract

**Background:**

Perivascular adipose tissue (PVAT), an active endocrine organ, exerts direct effect on vascular tone through paracrine. Activation of PVAT metabolism plays an inhibitory role in atherosclerosis *via* secreting relaxing factors. The present studies were designed to investigate the role of PVAT metabolism in regulation of hypertension.

**Materials and methods:**

Apolipoprotein E (ApoE) knockout mice with BMP4 knockout in adipose tissue or brown adipose tissue (aP2-DKO or UCP1-DKO, respectively) were used for exploring the role of impaired PVAT metabolism in hypertension. Vascular function was assessed using wire myography. The potential regulatory factor of vascular function was explored using qPCR and ELISA and further confirmed in perivascular fat cell line.

**Results:**

Knockout of BMP4 either in adipose tissue or specifically in BAT aggravates high-fat diet (HFD, 40% fat)-induced hypertension and endothelial dysfunction in ApoE^–/–^ mice. In the meanwhile, deficiency of BMP4 also aggravates Ang II (angiotensin II) -induced hypertension and vascular remodeling in ApoE^–/–^ mice. Moreover, deficiency of BMP4 inhibits NO release and induces ROS production. *In vitro* system, aortic rings pretreated with PVAT extracts from BMP4-DKO mice showed increased vasoconstriction and reduced endothelial-dependent relaxation compared with the controls. We further demonstrated that PVAT of BMP4-DKO mice expressed higher level of angiotensinogen (AGT) and Ang II compared with the controls.

**Conclusion:**

Impaired PVAT metabolism aggravates hypertension, and this effect is dependent on the activation of local renin-angiotensin-aldosterone system (RAAS). The results of this study first demonstrate the regulatory role of PVAT metabolism in hypertension.

## Graphical Abstract

BMP4 deficiency in PVAT promotes vascular remodeling and hypertension by activation of local RAAS. 
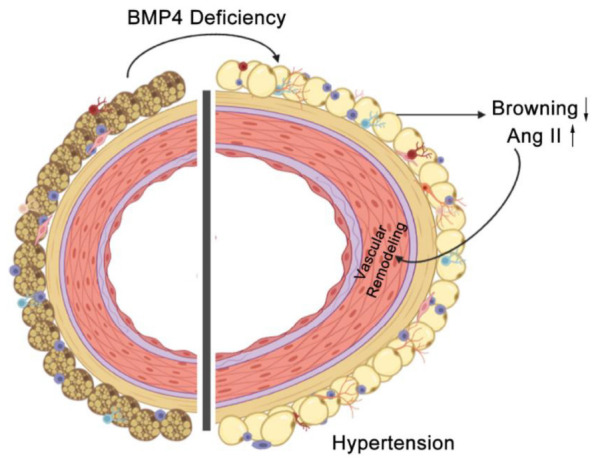

## Introduction

Hypertension, the main risk for myocardial infarction, stroke and coronary artery disease, is the leading contributing factor to global mortality (1, 2). Despite the various antihypertensive drugs have been used in clinical treatment, hypertension remains poorly controlled worldwide, and its prevalence is rising as the aging of population and the obesity epidemic (3). An analysis of the Framingham Heart Study reported a positive association between obesity and the relative risks of hypertension (4). In addition, excessive expansion and remodeling of adipose tissue during obesity significantly contributes to vascular dysfunction and cardiovascular diseases (5). Vascular remodeling, including endothelial dysfunction, increased vascular oxidative stress and increased perivascular inflammation and fibrosis is an important pathology in progression of hypertension (6–8).

Perivascular adipose tissue, the fat tissue surrounding most blood vessels, has attracted extensive attention as an active endocrine organ responsible for production and secretion of various bioactive factors that regulates vascular tone (9). In physiological conditions, PVAT produces protective bioactive factors such as nitric oxide (NO) and adiponectin which is helpful to regulate vascular tone and resist inflammation (10, 11). In pathological conditions, impaired PVAT secrets more detrimental bioactive factors and proinflammatory factors which induce vascular dysfunction and infiltration of inflammatory cells (8, 12), thus leading to hypertension. Due to PVAT’s property of proximity to blood vessels and function of regulating vascular homeostasis, PVAT has been considered as a potential target for improving vascular remodeling in hypertension. Mice lack of PVAT enhanced arterial stiffness in aging (13). Besides, removal of PVAT led to markedly enhanced neointimal hyperplasia in mice with endothelial injury (14). In contrast, activation of PVAT metabolism, characterized by more brown-like morphology and increased thermogenic activity, has beneficial impact on vascular remodeling. Mild cold exposure activates the thermogenic activity of PVAT, which contributes to protection against endothelial dysfunction (15). MitoNEET activates PVAT-dependent thermogenesis (16), and PVAT-specific overexpression of MitoNEET prevented arterial stiffness in mice (13). In addition, our previous study suggests that BMP4-mediated browning of PVAT prevents perivascular inflammation (17). Although PVAT metabolism is getting more attention in cardiovascular diseases, but it’s role in the development of hypertension remains unclear.

Renin-angiotensin-aldosterone system is a vital system for the body, as it contributes to the regulation of blood pressure (BP) and body fluid homeostasis (18). In clinical, many blockers in RAAS, including renin inhibitors, ACE inhibitors, and Ang II type 1 (AT1) receptor antagonists have been used in antihypertensive treatment (19). RAAS was firstly recognized as an endocrine system but important local forms of RAAS have been described in recent years (20). Most local RAAS are independent of the systemic RAAS and more directly involved in physiological and patho-physiological processes such as vascular remodeling and target-organ damage (21). Multiple lines of evidence suggest that hyperactivation of the local RAAS in the PVAT involves in the pathogenesis of cardiometabolic diseases (22), since most components of the RAAS have been detected in PVAT (23). Blood vessel rings pretreated with AT1R blockers markedly attenuated PVAT extracts-induced vasocontraction, suggesting that contractile effects of PVAT at least partly attributes to PVAT-derived Ang II (24). Additionally, brown adipocytes deficiency of AGT reduced the level of PVAT-derived Ang II and decreased BP in the resting phase, demonstrating that local RAAS in PVAT participates in the regulation of homeostatic circadian rhythmicity of BP (24). Lee et al. (25) demonstrated that PVAT-derived Ang1-7 induced relaxation of blood vessel by acting on the endothelium to induce the release of NO.

Bone morphogenetic protein 4 (BMP4), a member of transforming growth factor beta (TGFβ) superfamily, is closely associated with body metabolic homeostasis (26). Early studies indicate that serum BMP4 is markedly increased in non-diabetic individuals with obesity and metabolic syndrome (27). Peng et al. (28) demonstrated that overexpression BMP4 in liver prevented high-fat diet (HFD)-induced lipid accumulation in hepatocytes and alleviated the progression of non-alcoholic fatty liver disease (NAFLD). Overexpression of BMP4 in adipocytes promotes the browning of white adipose tissue (WAT), increases whole-body oxygen consumption and protects against HFD-induced metabolic disorders (29–31). In addition, our previous study demonstrated that BMP4 in PVAT exerts protective effects on atherosclerosis by activating PVAT metabolism (17), suggesting BMP4 -activated PVAT metabolism exerts beneficial effects on cardiovascular system. Thus, we hypothesized that BMP4 in PVAT is a critical mediator to maintain PVAT metabolism and protects against hypertension. In this study, we hypothesized that normal PVAT metabolism protects mice from hypertension, while impaired PVAT metabolism promotes the development of hypertension. To test this hypothesis, we used apolipoprotein E deficient (ApoE^–/–^) mice with adipose tissue BMP4 knockout or brown adipose tissue BMP4 knockout to examine effects of impaired PVAT metabolism on the development of hypertension. Here, we found that deficiency of BMP4 in PVAT accelerated obesity or Ang II-induced hypertension and vascular remodeling. Further, using *in vitro* experiments, we demonstrated that activation of local RAAS contributed to the impaired PVAT metabolism-related hypertension.

## Materials and methods

### Animals

Mice with adipose tissue-specific knockout of BMP4 and brown adipose tissue-specific knockout of BMP4 were generated by crossing BMP4^flox/flox^ mice with the aP2-Cre or UCP1- Cre knock-in mice and were subsequently crossed to the ApoE knockout mice to produce BMP4^Δ*aP*2^ ApoE^–/–^ (aP2-DKO), BMP4^Δ*UCP*1^ ApoE^–/–^ mice (UCP1-DKO), and littermate controls (the controls) (17) (Supplementary Figures 1A,B). 12-week HFD feeding (40% high-fat diet from Research Diets Inc., 12079B) of the BMP4-DKO mice (aP2-DKO and UCP1-DKO mice) induced hypertension development. Subcutaneous infusion of angiotensin II (Sigma-Aldrich, St. Louis, MO, USA) using osmotic mini-pumps (Alzet MODEL 1002; DURECT, Cupertino, CA, USA) at dose of 750 ng/kg/min for 14 days in BMP4-DKO mice at 10–12 weeks of age were used to develop hypertensive models. All studies using mice were approved by the Animal Care and Use Committee of the Fudan University Shanghai Medical College.

### Blood pressure measurement

The BP and HR of each mouse were measured by tail-cuff system (CODA, Kent Scientific, USA). At day time (1:00 p.m., to 3:00 p.m.), measurements were performed after 3 days of training. 20 constant measurements were recorded at each point (32). In Ang II-induced hypertensive mouse model, BMP4-DKO and the control mice were measured at −1, 2, 4, 6, 9, and 13 days before or after Ang II/PBS infusion.

### Vascular ring experiments

Carotid arteries were dissected free of PVAT and immediately placed in cold Krebs buffer (in g/L: NaCl 6.954, KCl 0.35, CaCl_2_⋅2H_2_O 0.368, MgSO4⋅7H_2_O 0.289, KH_2_PO_4_ 0.161, EDTA 0.01, Glucose 1.091, and NaHCO_3_ 2.1). The aortic rings were cut into 3 mm sections and suspended in an organ chamber containing 37°C Krebs buffer and bubbled with 95% O_2_ and 5% CO_2_. Aortic rings were connected to a force transducer to measure isometric tension. Next, the aortic rings need be equilibrated for 60 min under 2 mN tension. After equilibration and normalization procedures, aortic contractility was determined by addition of 60 mM KCl, phenylephrine, PVAT extracts, acetylcholine, and sodium nitroprusside.

### Nitric oxide (NO) and reactive oxygen species (ROS) evaluation

3-Amino,4-aminomethyl-2′,7′-difluorescein, diacetate (DAF-FM DA) (Catalog#S0019, Beyotime, China) and dihydroethidium (DHE) (Catalog#C1300-2, Applygen, China) were used to measure NO and ROS *in situ*, respectively, in aortic tissues of the control and BMP4-DKO mice. The procedures referred to methods of Victorio et al. (33). Briefly, aortic tissues were placed in Petri dishes with cold Krebs buffer and sectioned into 3-mm rings. For NO and ROS measurement, aortic rings were incubated 30 min with DAF-FM DA or DHE. Subsequently, aortic rings were fixed in 4% paraformaldehyde and embedded in freezing medium (Tissue-Tek, Sakura Finetek, USA) for getting frozen sections. images were obtained with a fluorescence microscope (Leica, Germany).

### Western blot

Western blot analyses were performed as previously described (17). Primary antibodies against the following proteins were used: eNOS (ABclonal, A20985, 1:1,000), phospho-eNOS (Ser1177) (Cell Signaling, 9571, 1:1,000), β-tubulin (ProteinTech Group, 66240-1-Ig, 1:10,000).

### Perivascular adipose tissue extracts

The procedures were same as described in works of Chang et al. (24). Briefly, the thoracic PVAT of mice with 12-week HFD were harvested and washed with cold PBS solution. Next, PVAT was homogenized in ice-cold PBS by a glass Dounce homogenizer to get extracts with a concentration of 1 ml/mg of volume/weight tissue. The well-homogenized extracts were centrifuged at 5,000 rpm for 10 min at 4°C to eliminate debris. At last, the liquid extracts were kept at 80°C for use.

### Quantitative PCR

Total RNA was extracted from PVAT or PV1 using the TRIzol (Life Technologies, Carlsbad, CA, USA) and stored DEPC H_2_O at −20°C. RNA quality and concentrations were determined using a microvolume spectrophotometer (Berthold, Germany). Then, using RevertAid First Strand cDNA Synthesis Kit (Thermo Fisher Scientific, USA), cDNA was synthesized from total 2 μg RNA. Next, the levels of multiple mRNAs, as indicated in the corresponding figures, were analyzed by qPCR using the primers shown in the Supplementary Table 1. The relative amount of each mRNA was calculated after normalization to the corresponding 18S mRNA, and the results were expressed as fold change relative to the control group.

### Measurement of perivascular adipose tissue angiotensinogen, and angiotensin II

Liquid extracts of PVAT were obtained as described in PVAT extracts section. PVAT AGT concentrations were quantitated using an ELISA kit (Catalog#27103, IBL, Japan). PVAT Ang II concentrations were quantitated using an ELISA kit (Catalog# CSB-E04495m, CUSABIO, China).

### Measurement of plasma angiotensinogen and angiotensin II

Plasma AGT concentrations were evaluated using an ELISA kit (Catalog#27103, IBL, Japan). Plasma AGT concentrations were evaluated using an ELISA kit (Catalog# E-EL-M2612c, Elabscience, China).

### Generation of immortalized perivascular fat cell

Perivascular adipose tissue were isolated from two 6-week wild-type (C57/BL6) mice, subsequently were minced and digested in digesting buffer (in g/L: NaCl 7.2, KCl 0.4, CaCl_2_ 0.14, Glucose 1, HEPES 23.8) containing 4% fatty acid-free bovine serum albumin (Sigma-Aldrich, USA) and 1 mg/ml collagnase (C2139, Sigma-Aldrich, USA) for 30 min at 37°C during gentle shaking. Next, the suspension was filtered through a cell strainer (40 μm size) and washed in DMEM/F12 by centrifugation for 200 g 5 min. Preadipocytes were resuspended and cultured in DMEM/F12 contain 10% fetal bovine serum (Gibco, USA), streptomycin (100 IU/mL) and penicillin (100 IU/mL).

For cell immortalization, preadipocytes at 80% confluence were infected with a retrovirus containing the plasmid, pBABE-neo sv40 large T (supplied by Dr. Dongning Pan, Fudan University). Subsequently, preadipocytes were cultured with DMEM/F12 containing G418 (1,000 ug/ml) for 2 days. After drug selection, individual cells were obtained by controlling dilution of cells into 96-well plates (0.2 cells in each well). With 7 to 10 days growth, each single cell cluster were digested and transferred to 48-well, 24-well, 12-well, 3.5, 6, and 10 mm plates. Cells in 10 mm plates were used for further *in vitro* study as passage 0.

### Culture and differentiation of immortalized perivascular fat cell

Immortalized perivascular fat cell (PV1) were plated and grown in DMEM medium supplemented with 10% fetal bovine serum (Gibco, USA), streptomycin (100 IU/mL) and penicillin (100 IU/mL). After reaching 80∼90% confluence (referred as day 0), preadipocytes were treated with induction cocktail (0.5 mM 3-isobutyl-1-methylxanthine, 1 μM dexamethasone, 10 μg/ml insulin, 50 nM T3, and 1 μM rosiglitazone) for 2 days. Then, cells were cultured in DMEM with differentiation cocktail (10 μg/ml insulin, 50 nM T3, and 1 μM rosiglitazone) for another 2 days. The medium was refreshed every other day. At day 8, mature adipocytes were harvested for further experiments.

### Statistical analysis

All data are shown as the mean ± SEM. GraphPad Prism 8.0 software (GraphPad Software, San Diego, CA, USA) was used to present the data. Statistical comparisons between two groups were performed by unpaired Student’s *t*-test, and comparisons among groups of three or more were performed by analysis of variance (ANOVA). In all statistical comparisons, a *p*-values of <0.05 was considered as a statistically significant difference. The numbers per group in the figure legends refer to the number of mice per group.

## Results

### Deficiency of bone morphogenetic protein 4 in perivascular adipose tissue aggravates high-fat diet-induced hypertension

**FIGURE 1:**
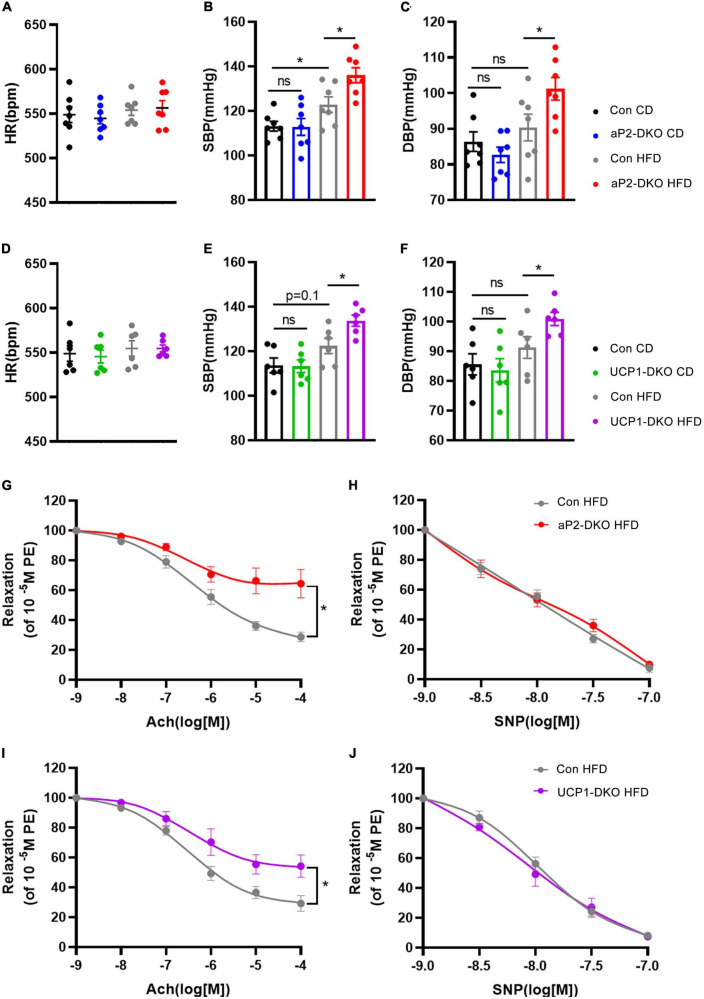
Deficiency of bone morphogenetic protein 4 (BMP4) in perivascular adipose tissue (PVAT) aggravates HFD-induced hypertension. Male the control, aP2-DKO and UCP1-DKO mice were fed with HFD for 12 weeks. **(A)** Heart rate. **(B)** Average systolic blood pressure (SBP) and **(C)** diastolic blood pressure (DBP). **(D)** Heart rate. **(E)** Average SBP and **(F)** DBP. **(G)** Concentration-response curves of endothelium-dependent (ACh) relaxation. **(H)** Concentration-response curves of endothelium-independent (SNP) relaxation. **(I)** Concentration-response curves of endothelium-dependent (ACh) relaxation. **(J)** Concentration-response curves of endothelium-independent (SNP) relaxation. Values are means ± S.E.M. *P < 0.05, **P < 0.01, and ***P < 0.001 by one-way analysis of variance (ANOVA) **(A–F)** or two-way analysis of variance (ANOVA) **(G–J)**.

Using two types of BMP4-DKO mouse models (Supplementary Figures 1A,B), our previous study found that deficiency of BMP4 in PVAT aggravated atherosclerosis development in ApoE^–/–^ mice. To evaluate the role of PVAT-derived BMP4 in hypertension, we measured the BP of two types of BMP4-DKO mouse models. In CD group, basal BP and HR were not different in aP2-DKO mice compared with the control mice (Figures 1A–C). HFD significantly increased systolic blood pressure (SBP) in control mice, and adipose tissue BMP4 knockout further promoted the elevation of SBP and diastolic blood pressure (DBP) (Figures 1B,C). Meanwhile, HFD and BMP4 knockout did not affect the HR (Figure 1A). Similarly, in CD group, there were no obvious differences in BP between UCP1-DKO and the control mice (Figures 1E,F). HR was not significantly influenced by diet and genotype (Figure 1D). Notably, the HFD-induced hypertension was further worsened in UCP1-DKO mice when compared with the controls (Figures 1E,F). These data suggested that deficiency of BMP4 in adipose tissue or brown adipose tissue both accelerates HFD-induced hypertension. Average SBP and DBP in aP2-DKO mice were 136.0 ± 3.4 and 101.2 ± 3.2 mm Hg, respectively (Figures 1B–C), modestly but not significantly higher than in UCP1-DKO mice (133.7 ± 2.5 and 100.9 ± 2.2 mm Hg, respectively; Figures 1E,F).

Vascular dysfunction has been observed in vascular remodeling and considered as an important contributor to hypertension. To further explore the impact of deficiency of BMP4 in PVAT on vascular function, we used intact carotid arteries isolated from the control mice and BMP4-DKO mice fed with HFD to examine endothelium-dependent and endothelium-independent relaxation responses. Interestingly, the endothelium-dependent relaxation induced by ACh was markedly attenuated in aP2-DKO group when compared with the control group, whereas no significant differences of endothelium-independent relaxation induced by SNP were observed between aP2-DKO group and the control group (Figures 1G,H). The same results were found in the UCP1-DKO mice (Figures 1I,J). Notably, we found that aP2-DKO group displayed worse endothelium-dependent relaxation (10^–4^ Ang II: 35.54% relaxation) compared with UCP1-DKO group (10^–4^Ang II: 45.74% relaxation). Together, these results suggested that deficiency of BMP4 in PVAT contributes to endothelial dysfunction.

### Deficiency of bone morphogenetic protein 4 in perivascular adipose tissue aggravates angiotensin II-induced hypertension

**FIGURE 2:**
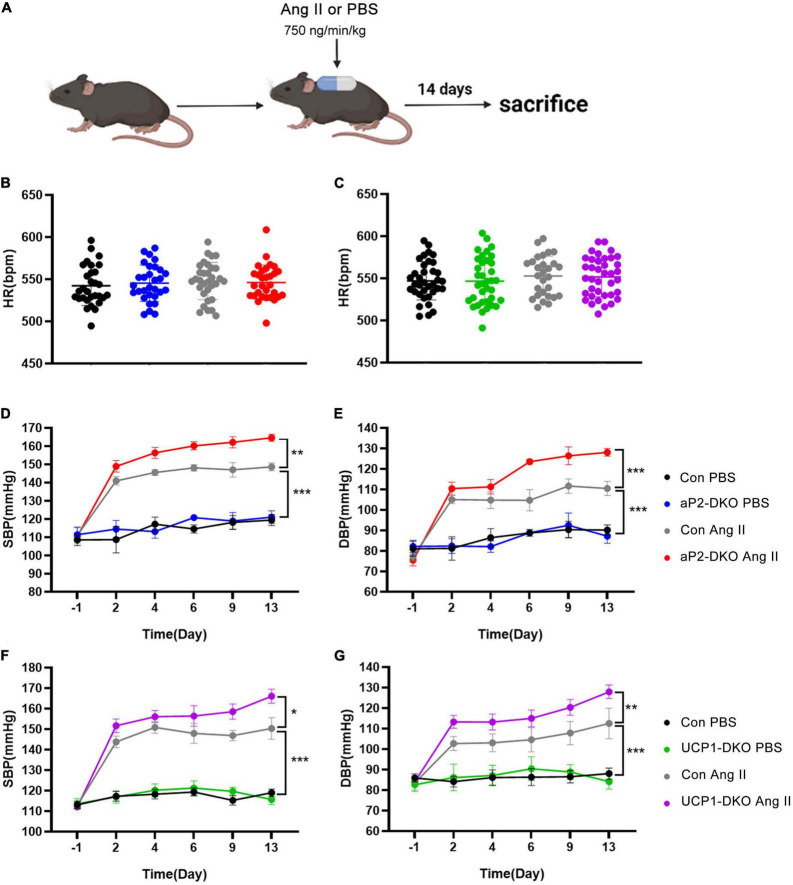
Deficiency of bone morphogenetic protein 4 (BMP4) in perivascular adipose tissue (PVAT) aggravates Angiotensin II-induced hypertension. **(A)** Animal models of Ang II (angiotensin II)-induced hypertension. **(B)** Heart rate of the control and aP2-DKO mice before and after Ang II/PBS administration. **(C)** Heart rate of the control and UCP1-DKO mice before and after Ang II/PBS administration. **(D)** Average SBP and **(E)** DBP of the control and aP2-DKO mice before and after Ang II/PBS administration. **(F)** Average SBP and **(G)** DBP of the control and UCP1-DKO mice before and after Ang II/PBS administration. Values are means ± S.E.M. *P < 0.05, **P < 0.01, and ***P < 0.001 by one-way analysis of variance (ANOVA) **(B,C)** or two-way analysis of variance (ANOVA) **(D–G)**.

Angiotensin II exerts its vasoconstrictor effect *via* its receptors on blood vessels. To further clarify the role of PVAT-derived BMP4 in the pathogenesis of hypertension, we treated two types of BMP4-DKO mouse with Ang II or PBS to generated Ang II–induced hypertensive model (Figure 2A). Despite no change in HR, Ang II-infused control mice exhibited markedly increase in BP compared with PBS-infused control mice (Figures 2B–G). Tail-cuff BP measurements revealed that adipose tissue BMP4 knockout did not affect the basal BP and HR (Figures 2B,D,E). Intriguingly, Ang II-induced a progressive increase of SBP and DBP was further increased in aP2-DKO mice compared with the controls (Figures 2D,E). Average SBP and DBP in aP2-DKO mice were significantly increased to 158.5 ± 1.6 and 120 ± 2.0 mmHg, respectively, markedly higher than in the control mice (145.9 ± 1.2 and 107.3 ± 1.6 mmHg, respectively). Similar changes of BP were observed in UCP1-DKO and the control mice (Figures 2C,F,G). In Ang II groups, brown adipose tissue BMP4 knockout further promoted the SBP and DBP elevation (Average SBP: control group: 147.9 ± 1.6 mmHg vs. UCP1-DKO group:157.0 ± 1.8 mmHg; Average DBP: control group: 105.8 ± 2.3 mmHg vs. UCP1-DKO group:116.9 ± 1.8 mmHg).

**FIGURE 3:**
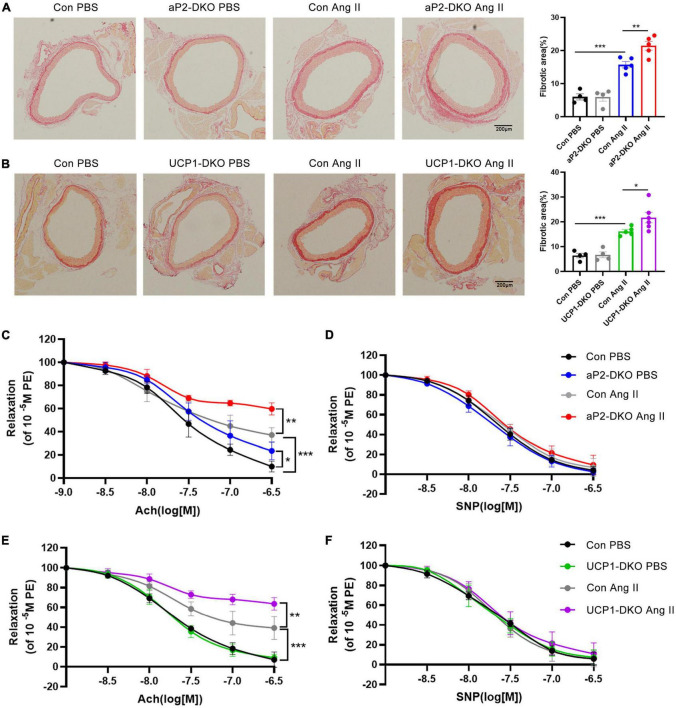
Deficiency of bone morphogenetic protein 4 (BMP4) in perivascular adipose tissue (PVAT) aggravates Ang II-induced vascular injury. Male the control, aP2-DKO and UCP1-DKO mice were infused with PBS or Ang II. **(A)** Sirius-red staining of thoracic aorta (left) and the percentage of fibrotic areas (right) were calculated. **(B)** Sirius-red staining of thoracic aorta (left) and the percentage of fibrotic areas (right) were calculated. **(C)** Concentration-response curves of endothelium-dependent (ACh) relaxation. **(D)** Concentration-response curves of endothelium-independent (SNP) relaxation. **(E)** Concentration-response curves of endothelium-dependent (ACh) relaxation. **(F)** Concentration-response curves of endothelium-independent (SNP) relaxation. Values are means ± S.E.M. *P < 0.05, **P < 0.01, and ***P < 0.001 by one-way analysis of variance (ANOVA) **(A,B)** or two-way analysis of variance (ANOVA) **(C–F)**.

Picrosirius red staining demonstrated that Ang II-induced aortic wall thickening and collagen deposition were drastically elevated in aP2-DKO and UCP1-DKO mice (Figures 3A,B). Then, we examined vascular functional changes. Carotid arteries isolated from BMP4-DKO mice exhibited more severe Ang II-induced endothelial dysfunction compared with that in the controls (Figures 3C,E). No significant changes were verified in SNP-induced relaxation between BMP4-DKO mice and the control mice (Figures 3D,F). Notably, adipose tissue BMP4 knockout reduced ACh-induced relaxation in PBS-infused mice, suggesting BMP4 knockout in WAT may contribute to endothelial dysfunction (Figure 3C). Taken together, these data demonstrated that deficiency of BMP4 in PVAT aggravated Ang II-induced hypertension and vascular dysfunction.

### Deficiency of bone morphogenetic protein 4 in perivascular adipose tissue inhibits nitric oxide release and induces reactive oxygen species production

**FIGURE 4:**
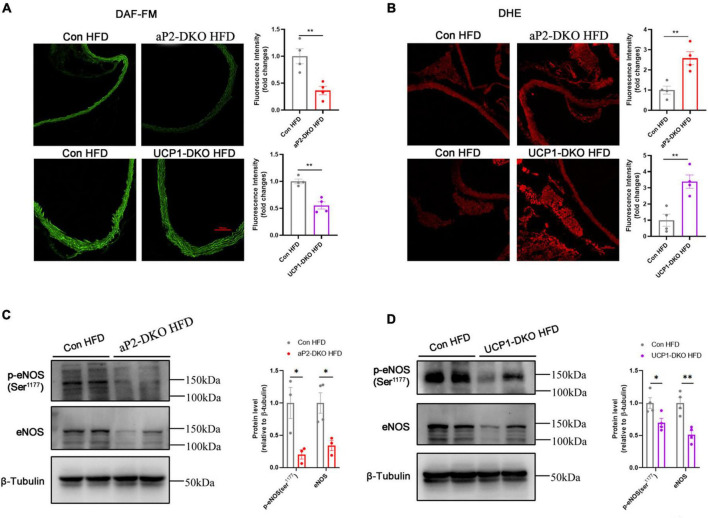
Deficiency of bone morphogenetic protein 4 (BMP4) in perivascular adipose tissue (PVAT) inhibits Nitric oxide (NO) release and induces reactive oxygen species (ROS) production. Aortic tissues were collected from the control, aP2-DKO and UCP1-DKO mice with 12-week HFD. **(A)** Representative fluorography for DAF-FM DA staining (left) and Quantification of DAF-FM DA fluorescent intensity (right) (*n* = 4). **(B)** Representative fluorography for DHE staining (left) and Quantification of DHE fluorescent intensity (right) (*n* = 4). **(C)** Western blot analysis (left) and quantification (right) of eNOS phosphorylation and total eNOS levels in aortae from the control and aP2-DKO mice. **(D)** Western blot analysis (left) and quantification (right) of eNOS phosphorylation and total eNOS levels in aortae from the control and UCP1-DKO mice. Values are means ± S.E.M. *P < 0.05, **P < 0.01, and ***P < 0.001 by unpaired Student’s t-test **(A–D)**.

The above data suggested that deficiency of BMP4 impaired endothelial function of ApoE^–/–^ mice. While endothelial-derived NO is mainly responsible for the regulation of endothelium on vascular tone (34). Vascular NO bioavailability affected by the expression of endothelial NO synthase and its activity. To further confirm the effect of BMP4 deficiency on endothelial function, we evaluated the vascular NO levels and found that the levels of NO in aortae of BMP4-DKO mice is obviously decreased compared with that in control mice (Figure 4A). Reactive oxygen species (ROS) can damage the endothelium and disrupt the balance of NO. Our results of dihydroethidium staining demonstrated that BMP4 deficiency dramatically induced ROS production (Figure 4B). Subsequently, we examined the level of total eNOS and phosphorylation of eNOS at Ser1177. Both total and phosphorylation of eNOS are decreased in aortae of BMP4-DKO mice compared with the controls (Figures 4C,D). Collectively, these results indicated that deficiency of BMP4 in PVAT inhibits NO release and induces ROS production.

### Bone morphogenetic protein 4-deficient perivascular adipose tissue induces vasoconstriction and endothelial dysfunction *in vitro*

**FIGURE 5:**
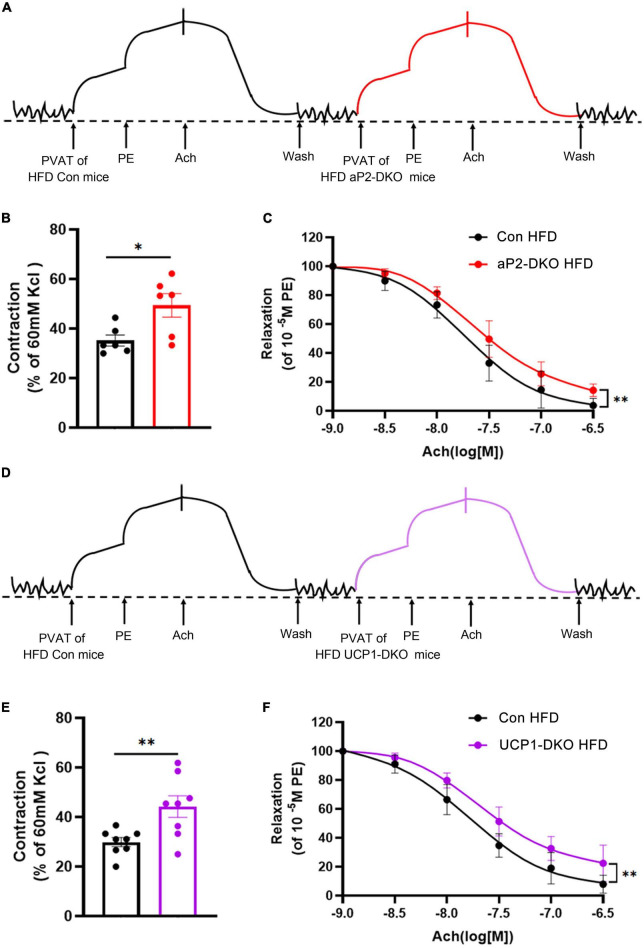
Bone morphogenetic protein 4 (BMP4)-deficient PVAT induces vasoconstriction and endothelial dysfunction *in vitro*. PVAT extracts were collected from the control, aP2-DKO and UCP1-DKO mice with 12-week HFD. **(A)** Representative trace showing aortic ring reaction in response to different treatments. **(B)** Quantitative analysis of constriction tension of aortic rings in response to PVAT extracts. **(C)** Vasodilator response induced by ACh in aortic rings treated with PVAT extracts. **(D)** Representative trace showing aortic ring reaction in response to different treatments. **(E)** Quantitative analysis of constriction tension of aortic rings in response to PVAT extracts. **(F)** Vasodilator response induced by ACh in aortic rings treated with perivascular adipose tissue (PVAT) extracts. Values are means ± S.E.M. *P < 0.05, **P < 0.01, and ***P < 0.001 by unpaired Student’s *t*-test **(B–E)** or two-way analysis of variance (ANOVA) **(C–F)**.

It’s well accepted that PVAT play a significant role in regulating vascular homeostasis *via* producing and secreting both dilative and constrictive factors according to the body’s needs. To define whether PVAT-derived BMP4 regulates vascular function by affecting PVAT-derived cytokines, we evaluated the vascular reactivity of wild-type aortic rings pretreated with PVAT extracts from BMP4-DKO mice or the controls fed with HFD for 12 weeks (Figures 5A,D). PVAT extracts from 10 mg PVAT of the control mice efficiently constricted the aortic rings from wild-type mice, and BMP4 knockout further promoted the constriction (Figures 5B,E). Then, we found that aortic rings pretreated with PVAT extracts from BMP4-DKO mice reduced ACh-induced relaxation (Figures 5C,F). Interestingly, the increased constriction induced by PVAT extracts from aP2-DKO mice is similar to that induced by PVAT extracts from UCP1-DKO mice (Figures 5B,E). These data indicate that PVAT derived BMP4 affects vascular function by regulating the paracrine of PVAT.

### Deficiency of bone morphogenetic protein 4 in perivascular adipose tissue activates local renin-angiotensin system

**FIGURE 6:**
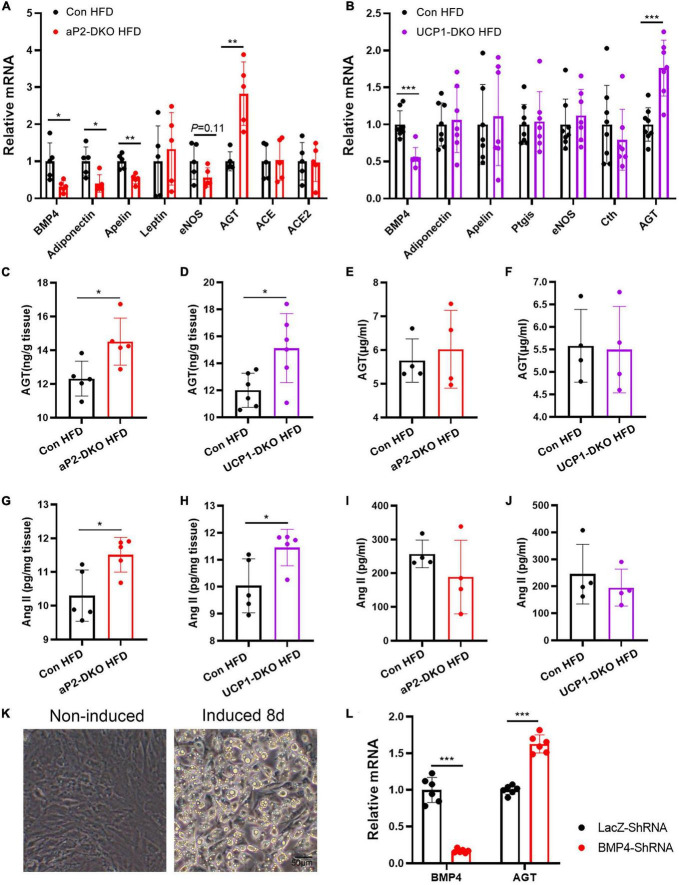
Deficiency of bone morphogenetic protein 4 (BMP4) in perivascular adipose tissue (PVAT) activates local renin-angiotensin system. **(A)** Relative mRNA levels of *BMP4*, *Adiponectin*, *Apelin*, *Leptin*, *eNOS*, *Cth*, *AGT*, *ACE*, and *ACE2* in PVAT of the control and aP2-DKO mice (*n* = 5). **(B)** Relative mRNA levels of *BMP4*, *Adiponectin*, *Apelin*, *Ptgis*, *eNOS*, *Cth*, and *AGT* in PVAT of the control and UCP1-DKO mice (*n* = 7–8). **(C)** AGT concentrations in PVAT from the control and aP2-DKO mice with 12-week HFD (*n* = 5). **(D)** AGT concentrations in PVAT from the control and UCP1-DKO mice with 12-week HFD (*n* = 6). **(E)** Plasma AGT concentrations in the control and aP2-DKO mice with 12-week HFD (*n* = 4). **(F)** Plasma AGT concentrations in the control and UCP1-DKO mice with 12-week HFD (*n* = 4). **(G)** Ang II concentrations in PVAT from the control and aP2-DKO mice with 12-week HFD (*n* = 5). **(H)** Ang II concentrations in PVAT from the control and UCP1-DKO mice with 12-week HFD (*n* = 5). **(I)** Plasma Ang II concentrations in the control and aP2-DKO mice with 12-week HFD (*n* = 4). **(J)** Plasma Ang II concentrations in the control and UCP1-DKO mice with 12-week HFD (*n* = 4). **(K)** Image of PV1 induced or non-induced to mature adipocytes. **(L)** Relative mRNA levels of *BMP4* and *AGT* in PV1 with BMP4 knockdown or lacZ knockdown. Values are means ± S.E.M. *P < 0.05, **P < 0.01, and ***P < 0.001 by unpaired Student’s *t*-test **(A–J,L)**.

Wong et al. (35) demonstrated exogenous BMP4 impaired endothelial-dependent relaxation, while we found that deficiency of BMP4 in PVAT also impairs endothelial-dependent relaxation, which means that BMP4 is not the factor that directly works on vascular function in our mouse models. To identify potential factors which could be responsible for vascular dysfunction, we examined mRNA expression of genes (adiponectin, apelin, leptin, eNOS, AGT, ACE, and ACE2), which have been proven with regulatory effect on vascular function, in PVAT of aP2-DKO and the control mice fed with HFD for 12 weeks. The gene expression of adiponectin, apelin, leptin, eNOS and AGT were remarkedly changed in in PVAT of aP2-DKO mice compared with the control mice (Figure 6A). To further determined which factor has a central role in PVAT BMP4 deficiency-induced vascular dysfunction, we examined mRNA expression of above genes and other genes with vascular-regulatory effect (Ptgis and Cth) in PVAT of UCP1-DKO and the control mice fed with HFD for 12 weeks. There was no significant difference in the expression of these genes, except AGT, which showed a 1.8-fold higher in UCP1-DKO group compared with the controls (Figure 6B). Further, we found significant increase in PVAT AGT concentrations of BMP4-DKO mice compared to the control mice (Figures 6C,D), while plasma AGT concentrations were not significantly different between mice of each genotype (Figures 6E,F). Therefore, we started to focus on the function of PVAT-derived AGT in hypertension.

As the only known precursor to Ang II, AGT is the most powerful biologically active products of the RAAS, almost involved in all of the classical actions of RAAS (18). Therefore, we sought to investigate whether the local RAAS is activated by impaired PVAT metabolism. As anticipated, PVAT concentrations of Ang II were significantly increased in two types of BMP4-DKO mice compared with the control mice (Figures 6G,H). However, plasma concentrations of Ang II did not show significant difference between BMP4-DKO and the control mice (Figures 6I,J). Growing data demonstrated adipose tissues, especially WAT, also are main source of AGT (36). To investigate whether WAT-derived BMP4 affects the expression of AGT in WAT, we examined the mRNA level of AGT in inguinal WAT (iWAT) and gonadal WAT (gWAT) of aP2-DKO and the control mice. Unlike in PVAT, deficiency of BMP4 did not enhance the expression of AGT in WAT, which might illustrate the result that aP2-DKO mice did not show higher BP than UCP1-DKO mice both in HFD-induced hypertensive and Ang II-induced hypertensive model (Supplementary Figures 2A,B). Taken together, these data suggest that local RAAS is important to the hypertension-protective effect of PVAT-derived BMP4.

White adipocytes are the main source of AGT in WAT. To find out whether increased AGT in dysmetabolic PVAT is arise from perivascular adipocytes, we constructed perivascular fat cell line PV1 and induced it to mature adipocytes (Figure 6K). PV1 highly expresses brown-related genes UCP1 compared with classical white fat cell 3T3-LI and immortalized brown preadipocytes PB (Supplementary Figure 3A), which suggested its thermogenic property. Next, BMP4 was knocked down in PV1 with a BMP4 shRNA. The results indicated that knockdown of BMP4 resulted in increased expression of AGT (Figure 6L). Therefore, these data support the idea that deficiency of BMP4 induced the expression of AGT in perivascular adipocytes, which is contribution to vascular dysfunction and hypertension.

## Discussion

Despite intensive studies on the protective effects of PVAT on cardiovascular system, the role of PVAT metabolism in the pathogenesis of cardiovascular diseases, especially in hypertension, which remains largely unknown. Our previous study indicates that loss of BMP4 in PVAT impaired PVAT metabolism and accelerated the development of atherosclerosis in ApoE^–/–^ mice (17). In this study, we provide novel evidence that impaired PVAT metabolism causes a marked exacerbation of hypertension and increases vascular dysfunction in two types of BMP4-DKO mice treated with HFD or Ang II, suggesting that activating PVAT metabolism may be an alternative novel approach for hypertension.

Obesity is often accompanied by several metabolic diseases, including hypertension. Data suggested that obese individuals have a 3.5-fold increased likelihood of having hypertension and 60% of hypertension is attributable to increased adipose stores (37). Both in obesity and hypertension, PVAT loses its protective and anti-contractile function (38). Previous studies suggested that HFD feeding markedly increased PVAT mass, resulting in whiting of PVAT, which indicates impaired PVAT metabolism (13, 14). Therefore, it is likely that PVAT metabolism is closely related to the development of obesity-induced hypertension. To investigate the role of PVAT metabolism in obesity-induced hypertension, we measured the BP of two-type BMP4-DKO and the control mice with fed with HFD. Our data suggests that impaired PVAT metabolism accelerated HFD-induced hypertension.

Hypertension is both a cause and a consequence of vascular remodeling (39). Blood vessels are sensitive to changes in extravascular environment, and make adaptive changes to maintain vascular homeostasis (40). However, in unhealthy states, these maladaptive changes cause vascular remodeling. In HFD-induced hypertensive mouse model, BMP4-DKO mice exhibited impaired endothelium-dependent vasodilation, reduced endothelial NO release and increased vascular ROS production. Besides, in Ang II-induced hypertensive mouse model, BMP4-DKO mice showed further endothelial dysfunction, increased intima-media thickness and collagen deposition. These results suggested that impaired PVAT metabolism leads to vascular remodeling. Extensive studies found that PVAT actively regulates blood vessel tone *via* producing and secreting vasoactive factors, including both relaxing and contracting factors (41). To focus on the direct effects of dysmetabolic PVAT on vascular function, we treated aortic rings of wild-type mice with PVAT extracts from BMP4-DKO mice and the control mice. Our data demonstrated that dysmetabolic PVAT further induces vasoconstriction and leads to endothelial dysfunction. Therefore, these findings suggest that PVAT-derived vasoactive factors have a central role in PVAT metabolism-regulated hypertension.

Renin-angiotensin-aldosterone system hyperactivity is one of the pivotal mechanisms mediating cardiovascular diseases (42). Increasing evidence have proved the presence of local RAAS activity in PVAT (43). Obesity results in increased production of AGT, Ang II and aldosterone in PVAT (43). These factors induce vasocontraction, oxidative stress and arterial stiffening (44, 45). However, Costa et al. demonstrated that 4-week high-carbohydrate diet enhanced the anticontractile effect of PVAT by activating local RAAS, emphasizing the compensatory adaptive characteristic of PVAT to preserve the vascular function in initiation of obesity (46). Herein, we showed that deficiency of BMP4 in PVAT activated local RAAS and enhanced PVAT-derived Ang II, which demonstrated a regulatory effect of PVAT metabolism on local RAAS. Using perivascular fat cell line, we further demonstrated the central role of perivascular adipocyte-derived AGT in PVAT metabolism-mediated local RAAS.

Here, we used two different BMP4 knockout mouse models (in adipose tissue or BAT) to examine whether changes of PVAT metabolism, regulated by BMP4, could have an effect on hypertension development. Compared to UCP1-DKO mice, aP2 DKO mice have BMP4 knockout in WAT. However, WAT also has an effect on cardiovascular system. As an endocrine organ, WAT can regulate cardiovascular system by producing distinct cytokines under physical and pathological conditions (47, 48). In addition, several studies have demonstrated the exist of local RAAS in WAT (36, 49). Retroperitoneal adipose tissue even is one of the primary sites of systemic AGT (50). Mice with transgenic overexpression of AGT in adipocytes exhibits increased plasma AGT concentrations and SBP (51). Notably, aP2-Cre-mediated deficiency of AGT in adipocytes reduced plasma Ang II concentrations and decreased BP in male fed a high-fat diet (52). Our results showed that deficiency of BMP4 in PVAT enhanced the level of AGT in PVAT, while deficiency of BMP4 in WAT did not influence the level of AGT in WAT. In addition, two types of BMP4-DKO mice did not show obvious difference in plasma concentrations of AGT and Ang II compared with the control mice. These data suggest that deficiency of BMP4 did not activate the local RAAS in WAT and circulating RAAS. Interestingly, we observed that aP2-DKO exhibited slightly impaired endothelium-dependent vasodilation in physiological condition, and showed more severe endothelial dysfunction compared with UCP1-DKO mice in HFD condition, but we did no find more severe vascular remodeling and higher BP in aP2-DKO compared with UCP1-DKO mice in pathological condition. We do find difference in expression of vasoactive factors in PVAT of aP2-DKO and UCP1-DKO mice. To focus on the role of PVAT metabolism in hypertension development, we chose AGT for further study. The expression changes of AGT, Apelin and eNOS in aP2-DKO mice may results from higher knockout efficacy of aP2-Cre or indirect effect from WAT. To answer this question, we need further study.

This study investigated the role of PVAT metabolism in the differential regulation of BP. The major findings of these studies are (1) impairing BMP4-mediated PVAT metabolism further increased BP in mice with obesity or Ang II-induced hypertension, (2) PVAT metabolism has direct effect on vascular function, (3) PVAT metabolism involves in the regulation of local RAAS activity. These data demonstrate a role for activation of PVAT metabolism in protection of hypertension and provide a novel approach to the management of hypertension.

## Conclusion

We demonstrated that mice with deficiency of BMP4 in PVAT aggravates hypertension development. It was also observed that PVAT of BMP4-deficient mice have higher level AGT and Ang II than the controls. These indicates that BMP4 in PVAT has a protective role in hypertension development *via* regulating local RAAS.

## Data availability statement

The original contributions presented in this study are included in the article/Supplementary material, further inquiries can be directed to the corresponding authors.

## Ethics statement

The animal study was reviewed and approved by the Animal Care and Use Committee of the Fudan University, Shanghai Medical College.

## Author contributions

YT, W-JM, and Q-QT designed the experiments and supervised the study. W-JM, Y-JS, S-WQ, and L-JY carried out the animal and molecular experiments. W-JM, YT, Q-QY, and YL analyzed the data. W-JM and Y-JS wrote the original draft. YT and Q-QT reviewed and edited the manuscript. All authors contributed to the article and approved the submitted version.
