# Supplementary material for: Bone morphogenetic protein 4 in perivascular adipose tissue ameliorates hypertension through regulation of angiotensinogen
Source: Front Cardiovasc Med. 2022 Nov 14;9:1038176. doi: 10.3389/fcvm.2022.1038176 (PMC9707298; doi:10.3389/fcvm.2022.1038176)
Supplement: Supplementary file 1 [file Data_Sheet_1.docx]

Supplementary Materials for

**BMP4 in perivascular adipose tissue ameliorates hypertension through regulation of angiotensinogen**

Wen-Juan Mu^1,^ ^†^, Yan-Jue Song ^1, †^, Li-Jie Yang^1^, Shu-Wen Qian^1^, Qi-Qi Yang^1^, Yang Liu^1^, Qi-Qun Tang^1,*^, Yan Tang^1,*^

**This PDF file includes:**

Supplementary Figures 1 - 3

Supplementary Tables 1


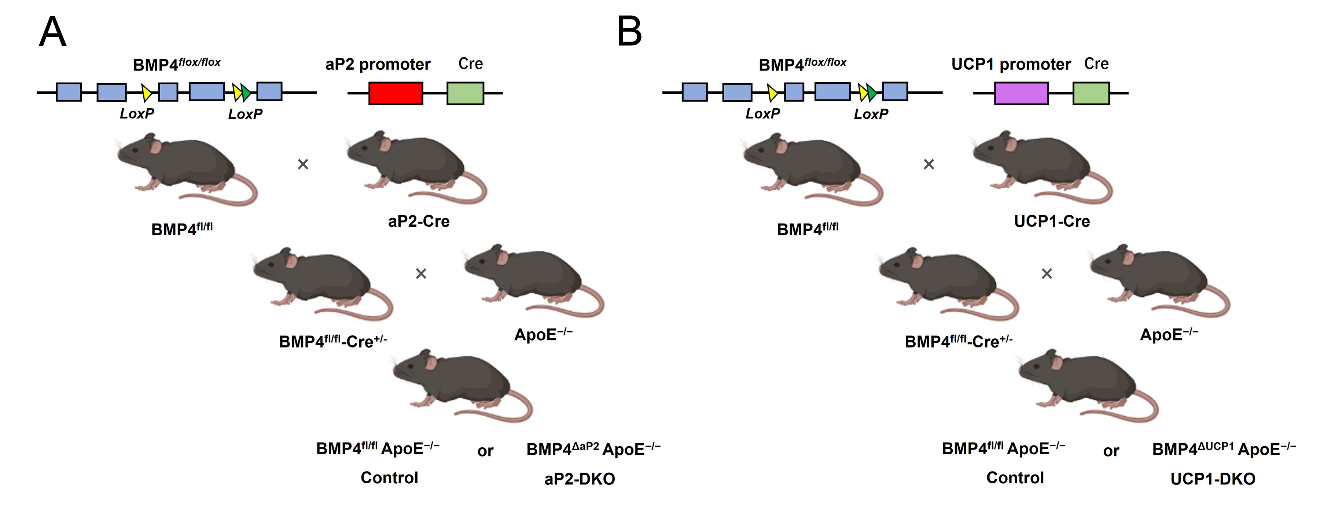
**Fig. S1.**

**Figure S1. Schematic illustration of BMP4-DKO mouse models.** (A) Generation of aP2-DKO mouse model. (B) Generation of UCP1-DKO mouse model.

**Fig. S2.**


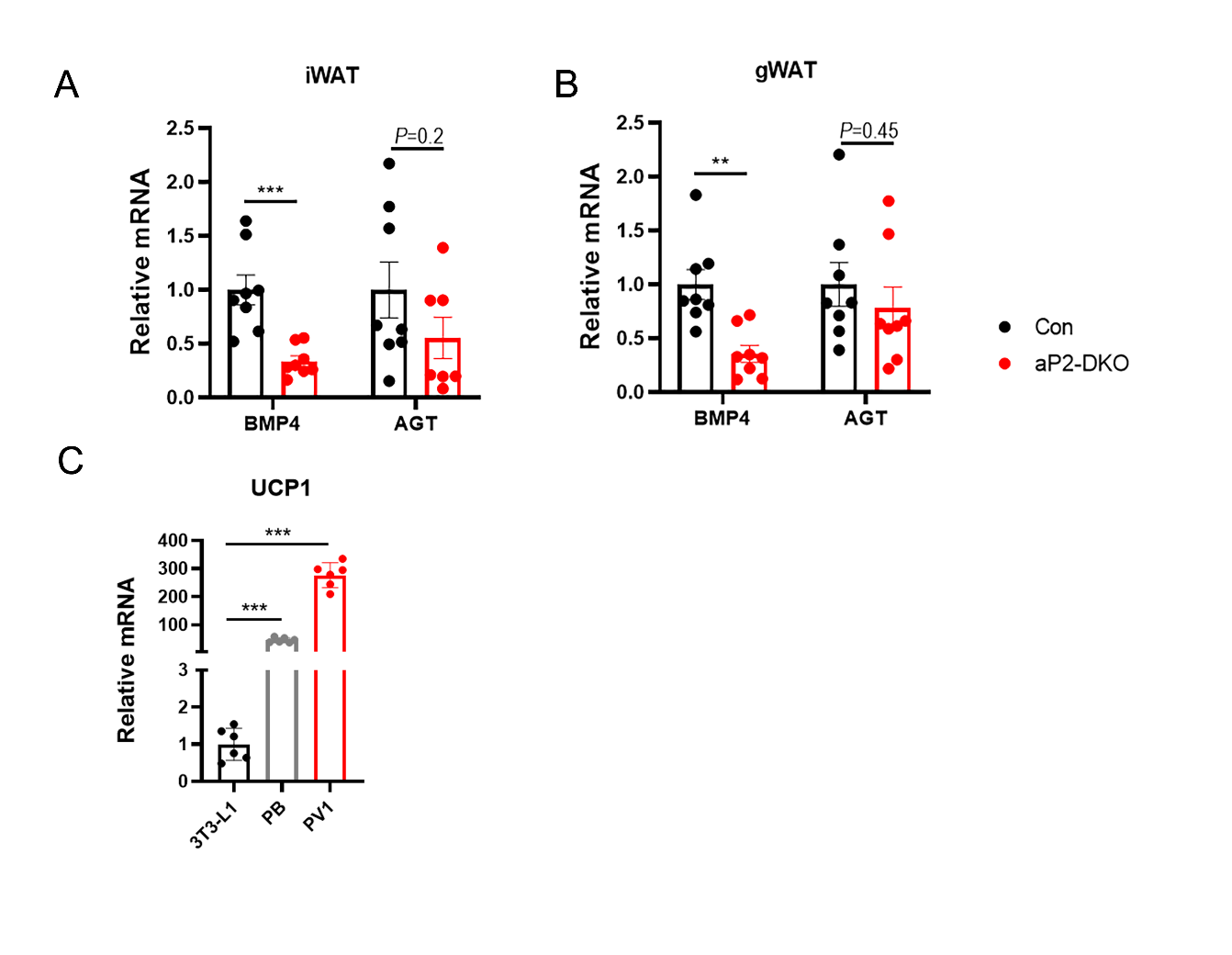


**Figure S2.** **BMP4 deficiency did not affect the level of AGT in WAT.** (A) Relative mRNA levels of *BMP4* and *AGT* in iWAT of the control and aP2-DKO mice with HFD for 12 weeks (n=7-8). (b) Relative mRNA levels of *BMP4* and *AGT* in gWAT of the control and aP2-DKO mice with HFD for 12 weeks (n=7-8). Values are means±S.E.M. *P<0.05, **P<0.01, ***P<0.001 by unpaired Student’s t test (A and B).

**Fig. S3.**


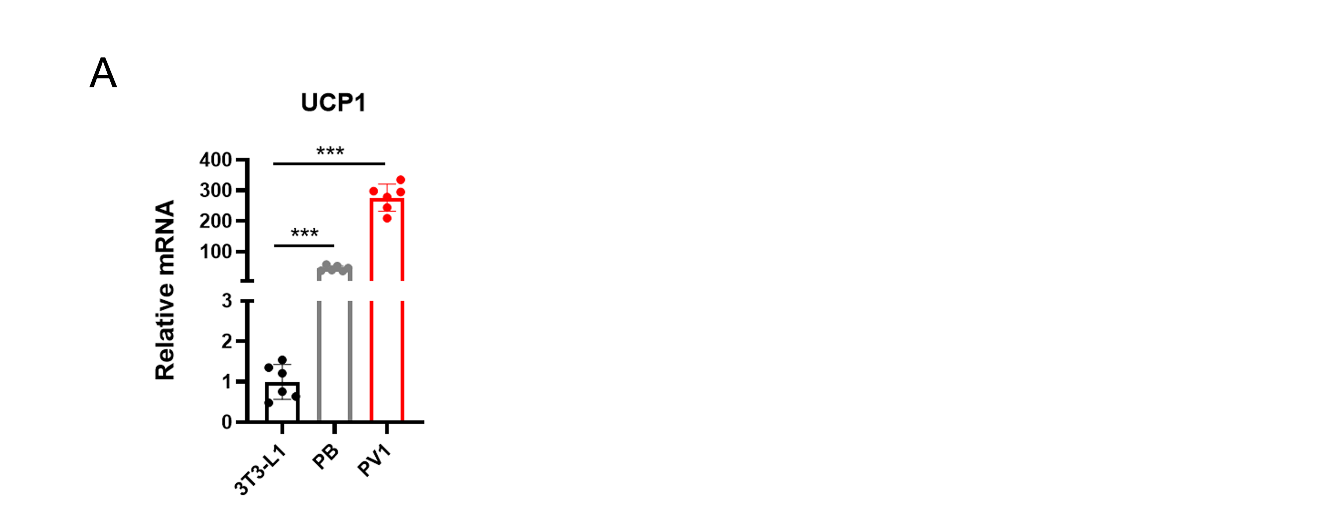


**Figure S3.** **Gene expression of UCP1 in different fat cell lines.** (A) Relative mRNA levels of *UCP1* in 3T3-L1, mature brown adipocyte (PB) and mature perivascular adipocyte (PV1). Values are means±S.E.M. *P<0.05, **P<0.01, ***P<0.001 by one-way analysis of variance (ANOVA) (A).

**Table S1. Primers for qPCR used in this study.**

|  | **Forward** | **Reverse** |
| --- | --- | --- |
| **Mouse UCP-1** | AGGCTTCCAGTACCATTAGGT | CTGAGTGAGGCAAAGCTGATTT |
| **Mouse Ptgis** | ACAGCATCAAACAATTTGTCGTC | GCATCAGACCGAAGCCATATCT |
| **Mouse cth** | TTCCTGCCTAGTTTCCAGCAT | GGAAGTCCTGCTTAAATGTGGTG |
| **Mouse ACE2** | GCAGATGGCTACAACTATAACCG | CCTCCTCACATAGGCATGAAGA |
| **Mouse ACE** | CCACCAGGGCCCACTACACC | GACTTCGCCATTCCGCTGATT |
| **Mouse AGT** | GCGGAGGCAAATCTGAACAACAT | GAAGGGGCTGCTCAGGGTCACAT |
| **Mouse eNOS** | TGTGACCCTCACCGCTACAA | GCACAATCCAGGCCCAATC |
| **Mouse leptin** | GAGACCCCTGTGTCGGTTC | CTGCGTGTGTGAAATGTCATTG |
| **Mouse Adiponectin** | TGTTCCTCTTAATCCTGCCCA | CCAACCTGCACAAGTTCCCTT |
| **Mouse Apelin** | TCTTGGCTCTTCCCTCTTTTCA | GTGCTGGAATCCACTGGAGAA |
| **Mouse BMP4** | TTCCTGGTAACCGAATGCTGA | CCTGAATCTCGGCGACTTTTT |
| **Mouse 18s** | CGCCGCTAGAGGTGAAATTCT | CATTCTTGGCA1AATGCTTTCG |
